# Identification and Conservation Analysis of *Cis*-Regulatory Elements in Pig Liver

**DOI:** 10.3390/genes10050348

**Published:** 2019-05-07

**Authors:** Yu Luan, Lu Zhang, Mingyang Hu, Yueyuan Xu, Ye Hou, Xinyun Li, Shuhong Zhao, Yunxia Zhao, Changchun Li

**Affiliations:** 1 Key Laboratory of Agricultural Animal Genetics, Breeding, and Reproduction of the Ministry of Education and Key Laboratory of Swine Genetics and Breeding of Ministry of Agriculture, Huazhong Agricultural University, Wuhan 430070, China; ly178574378@hotmail.com (Y.L.); luzhang@webmail.hzau.edu.cn (L.Z.); myhu@webmail.hzau.edu.cn (M.H.); yyxu@webmail.hzau.edu.cn (Y.X.); houye2009@126.com (Y.H.); xyli@mail.hzau.edu.cn (X.L.); shzhao@mail.hzau.edu.cn (S.Z.); 2 The Cooperative Innovation Center for Sustainable Pig Production, Wuhan 430070, China

**Keywords:** *cis*-regulatory elements, pig liver, conservation

## Abstract

The liver plays a key role in metabolism and affects pig production. However, the functional annotation of noncoding regions of the pig liver remains poorly understood. We revealed the landscape of *cis*-regulatory elements and their functional characterization in pig liver. We identified 102,373 *cis*-regulatory elements in the pig liver, including enhancers, promoters, super-enhancers, and broad H3K4me3 domains, and highlighted 26 core transcription regulatory factors in the pig liver as well. We found similarity of *cis*-regulatory elements among those of pigs, humans, and cattle. Despite the low proportion of functionally conserved enhancers (~30%) between pig and human liver tissue, ~78% of the pig liver enhancer orthologues sequence could play an enhancer role in other human tissues. Additionally, we observed that the ratio of consistent super-enhancer-associated genes was significantly higher than the ratio of functionally conserved super-enhancers. Approximately 54% of the core regulation factors driven by super-enhancers were consistent across the liver from these three species. Our pig liver annotation and functional characterization studies provide a system and resource for noncoding annotation for future gene regulatory studies in pigs. Furthermore, our study also showed the high level functional conservation of *cis*-regulatory elements in mammals; it also improved our understanding of regulation function of mammal *cis*-regulatory elements.

## 1. Introduction

Several years after the complete genome sequencing, scientists have annotated the functional genomes of different species, including human [1,2,3], mice [4,5], *Drosophila melanogaster* and *Caenorhabditis elegans* [6,7,8]. Human disease research has been rapidly improved by these studies. Based on the *cis*-regulatory elements identified by functional genomes, most variants of *cis*-regulatory elements are believed to offer risks to various common human diseases [9,10]. Resources, such as Encyclopedia of DNA Elements (ENCODE) and Roadmap Epigenome Project, have shown that variants of *cis*-regulatory elements are related to physiological traits and diseases at considerable genome-wide association values [1,2,3]. Therefore, human disease research has been rapidly improved by these studies.

Promoters and enhancers are two types of *cis*-regulatory non-coding sequences that have major roles in transcriptional regulation during cell development [11,12]; moreover, they have important evolutionary roles as well [13]. The H3K4me3 and H3K27ac are two primary histone-modification markers and can identify enhancers and active promoters [14]. Recent studies have suggested that the regions with extensive enrichment of histone modification are associated with cell identity and disease [15,16,17]. Super-enhancers are large enhancer regions that drive cellular development and depend on cell identity genes [15,16], and can be identified by H3K27ac ChIP-seq intensity. Additionally, the broad H3K4me3 domain has been reported to increase transcription elongation and has been associated with disease-associated genes [17,18].

Enhancers display rapid evolution across many species [14], and several studies have reported highly regulatory elements of orthologous sequences between species that are still functionally conserved cross lineage–species [5,19]. Genome-wide analysis of domesticated species and genome-wide-associated study of livestock have produced numerous notable loci that are related to domestication [20,21,22]. Most of these loci are located in noncoding regions [23,24], and this finding is consistent with human studies.

Pigs are important in agriculture industry and increasing studies tend to use pigs as biomedical models [25,26]. The liver governs numerous physiological processes, especially regarding metabolism, and it drives feeding efficiency, growth, etc. economical traits in pigs [27,28,29]; however, the *cis*-regulatory elements, especially enhancers, active promoters, super-enhancers, and broad H3K4me3 domain, in pig liver tissue remain unknown. The ongoing Functional Annotation of Animal Genomes project (FAANG) in livestock aims to provide comprehensive maps of functional elements in the genomes of domesticated animal species [30].

We systematically identified the *cis*-regulatory elements, including enhancers, promoters, super-enhancers, broad H3K4me3 domain, and core transcription regulatory factors, in the pig liver tissue by using ChIP-seq data [14]. Moreover, these *cis*-regulatory elements were related to the physiological and metabolic processes of the liver. We compared the characteristics of *cis*-regulatory elements among pigs, humans, and cattle, and found that a certain number of elements are conserved between these three species.

## 2. Materials and Methods

### 2.1. ChIP-Seq Data Processing

We downloaded three-species H3K27ac and H3K4me3 ChIP-seq data from Villar et al. [14]. All work presented in this paper was based on susScr11, hg38 and bosTau8 reference genome sequence data from the UCSC Genome Browser. BWA v0.7.13 was used to align the sequencing data to the reference genome [31]. Reads with low-mapping quality scores were removed (MAPQ < 25). The PCR duplication reads were removed by using Picard v1.126 (Broad Institute of MIT and Harvard, Cambridge, USA).

To check repeatability between biological replicates, we divided the reference genomes into 10 kb bins and computed the number of reads within each bin. The Pearson correlation coefficient between each biological replication for H3K4me3 and H3K27ac in the three species were calculated by using above-normalized 10 kb bins reads. After confirming that all replicates were highly correlated, we pooled their bam files together by using the merge function of SAMTOOLS for further analysis.

The MACS v2.1.0 peak caller [32] was used to compare ChIP signals to a corresponding DNA input to identify narrow peaks. Narrow peaks with Poisson *p*-values greater than 0.01 and broad peaks with Poisson *p*-value greater than 0.1 were removed to ensure good quality peaks for further analysis. Reads per million (RPM) of IP data and input data in each peak region were calculated, and the qualified peaks should pass the threshold of two-fold enrichment (RPM_IP_ ≥ 2 × RPM_Input_) and RPM_IP_-RPM_Input_ >1. The genome-wide signal coverage tracks were generated by using MACSv2.10 peak caller for each histone ChIP-seq data.

### 2.2. RNA-Seq Data Analysis

We downloaded three-species RNA-seq data from Berthelot et al. [33], which is consistent with ChIP-seq and generated by the same research group. STARv2.5.3 (Cold Spring Harbor Laboratory, Cold Spring Harbor, NY, USA) was used to align RNA-seq data to reference genomes with “outSAMtype BAM SortedByCoordinate-quantMode TranscriptomeSAM” parameters. The genome-wide signal coverage tracks were generated by using STARv2.5.3 with the “-outWigStrand Stranded” parameter. RSEM (https://github.com/deweylab/RSEM) was used to quantify and calculate the expression values.

### 2.3. Identification of Cis-Regulatory Elements

The promoters were identified by using H3K4me3 peaks. Among them, the promoters with H3K27ac peaks were defined as active promoters. The H3K27ac peak regions without H3K4me3 peaks and outside of 2.5 kb upstream and 1.5 kb downstream of transcription start sites (TSS) were identified as enhancers. The super-enhancers were identified by using ROSE (https://bitbucket.org/young_computation/rose) with default parameters [15,34]. The H3K4me3 peaks were inside of 2.5 kb upstream and 1.5 kb downstream of TSS (transcription start site), and the widths greater than 4 kb were identified as broad H3K4me3 domains. To ensure we had good quality *cis*-regulatory elements and their consistence were identified for the further analysis, we span the 2000 base pairs for each element. Then, we computed the RPM value for the 2 kb region centered at the “summit” of each *cis*-regulatory element. The qualified elements should pass the threshold of two-fold enrichment (RPM_IP_ ≥ 2 × RPM_Input_) and RPM_IP_-RPM_Input_ > 1. Core transcription regulatory factor was identified by using CRCmapper [35] pipeline, which included the bamToGFF tools and fimo software.

### 2.4. Identification of Functionally Conserved Cis-Regulatory Elements

All *cis*-regulatory elements were unified to the size of 2 kb according to peak summits. We converted *cis*-regulatory elements in susScr11 to hg38 and bosTau8 coordinates by using UCSC LiftOver tools (https://genome.ucsc.edu/cgi-bin/hgLiftOver) with a minimum match of 0.2. We identified functionally conserved elements in pigs that have orthologous *cis*-elements in human and cattle liver tissues. The ROADMAP epigenomics project chromatin imputed data were downloaded from https://egg2.wustl.edu/roadmap/.

### 2.5. Differential Expression Analysis

To normalize the gene expression levels between different species, we used the quantile normalization function in R. Moreover, edgeR was used to identify differential expression (DE) orthologous genes between the pigs, humans, and cattle liver samples [36]. The “one2one” orthologous genes showing |log_2_Fold| ≥ 2, *p*-value < 0.001 and fals− discovery rate (FDR) <0.01 were considered to be differentially expressed.

### 2.6. Motif Analysis and Functional Enrichment Analysis

We performed motif analysis by using Homer findMotifsGenome [36] on the *cis*-regulatory element regions with default parameters. The function enrichment analysis using GREAT software.

## 3. Results

### 3.1. Identification of Cis-Regulatory Elements in Pig Liver Tissue

**Figure 1:**
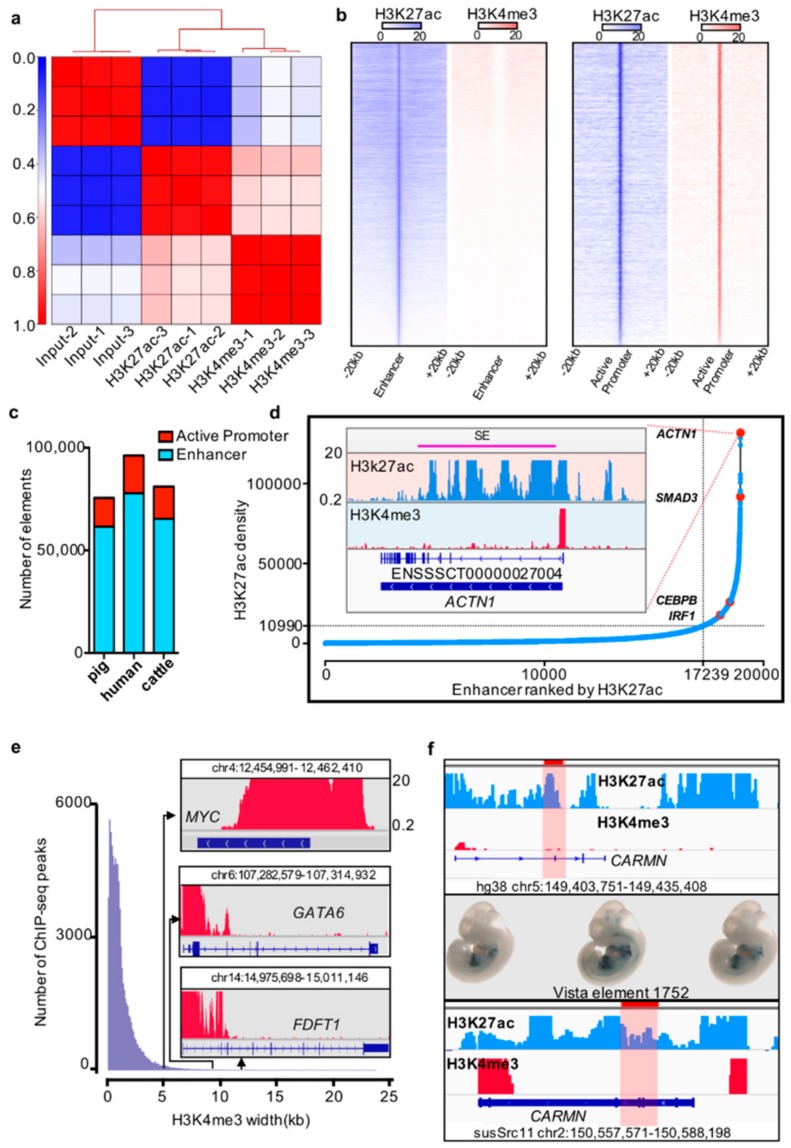
*Cis*-regulatory elements in pig liver tissue. (**a**) Correlation between biological replicates for H3K27ac and H3K4me3 histone ChIP-seq (pig). (**b**) Heatmap of the enhancer and active promoter signals. Blue and red represent the H3K27ac and H3K4me3 signal levels, respectively. (**c**) Number of active promoters and enhancers in pigs, humans, and cattle. (**d**) Distribution of H3K27ac signal at enhancers. The number of ranked typical enhancers and super-enhancers by H3K27ac density (*x*-axis) and the density of H3K27ac (*y*-axis) are plotted. The crossline represents the density cutoff used for identifying the super-enhancers. The red dot represents four examples of super-enhancer-associated genes. The H3K27ac and H3K4me profiles of the *ACTN1* gene are indicated in the inserted box. (**e**) Width distribution of H3K4me3 ChIP-seq peaks. Inset: example of H3K4me3 signals of *MYC*, *GATA*6, and *FDFT1* promoter regions. (**f**) Example of pig enhancer conserved with humans and overlapping with the VISTA Enhancer Browser. The first box shows the human enhancer element 1752 with H3K4me3 and H3K27ac profiles in liver tissue; the highlight represents the element 1752 loci. The second box represents the element 1752 expression pattern from the VISTA Enhancer Browser. The third box exhibits the Genome Browser screenshot of the conserved elements in pig liver tissue.

To identify the *cis*-regulatory elements in pig liver tissue, we analyzed ChIP-seq data by using two critical histone markers (H3K4me3 and H3K27ac) for promoter and enhancer prediction [14,37] (Supplementary Materials Table S1). The Pearson correlation coefficient between biological replicates was calculated, and biological replicates exhibited high correlation with each other (R > 0.9) (Figure 1a, Supplementary Materials Table S2). The replicates for each histone ChIP-seq data were pooled for future data analyses (Supplementary Materials Table S3). We used the enrichment of H3K4me3 signals to predict promoters, H3K4me3 signals overlapped with H3K27ac signal to predict active promoters, and H3K27ac signals outside of promoters and TSS regions to predict enhancers [4,14] (Figure 1b). We identified 14,007 active promoters out of 37,103 promoters and 61,570 enhancers in pig liver tissue (Figure 1c; Supplementary Materials Table S4).

In previous studies, super-enhancers and broad H3K4me3 domains were reported to have important roles in the maintenance of cell identity and cell development, as well as controlling disease-related genes [15,38]. We identified the super-enhancers and broad H3K4me3 domains by using the ROSE pipeline and a previously described method [15,17,34,38]. We identified 1711 super-enhancers and 1989 broad H3K4me3 domains in the pig liver (Figure 1d,e; Supplementary Materials Table S5).

Previous studies proposed that functional elements are partially conserved [5,13,14,39]. To further explore the conservation of functional elements between pigs and other mammals, especially the special regulatory elements, such as super-enhancers and broad H3K4me3 domains, we analyzed the liver ChIP-seq data of humans and cattle by using the same method as above to identify *cis*-regulatory elements (Figure 1C, Supplementary Materials Table S5). The enhancers located in the *CARMN* gene body region in the pig are sequence conserved with hs1752 elements in humans, and it plays an enhancer role in human liver tissue. We also noted that this enhancer expressed in mouse heart and liver at E11.5 day in the VISTA Enhancer Browser [40] (Figure 1f).

### 3.2. Conservation of Cis-Regulatory Elements 

The observation of conserved functional elements in pigs, humans, and cattle prompted us to investigate the conservation of these elements across evolution. Thus, we compared different types of functional elements using UCSC LiftOver. The results indicated that a majority of pig promoters are conserved with humans and cattle. In comparison with human promoters, 90% of pig promoters were sequence conserved, and 73.7% played promoter roles in humans. The number of promoters between pigs and cattle was 86.1% and 67.7%, respectively. The same conservation analysis of active promoters showed that 96.1% and 91.4% of pig active promoters were sequence conserved with humans and cattle, respectively. Moreover, 77.5% and 76.4% of active pig promoters were conserved and functioned as active promoters in humans and cattle, respectively.

**Figure 2:**
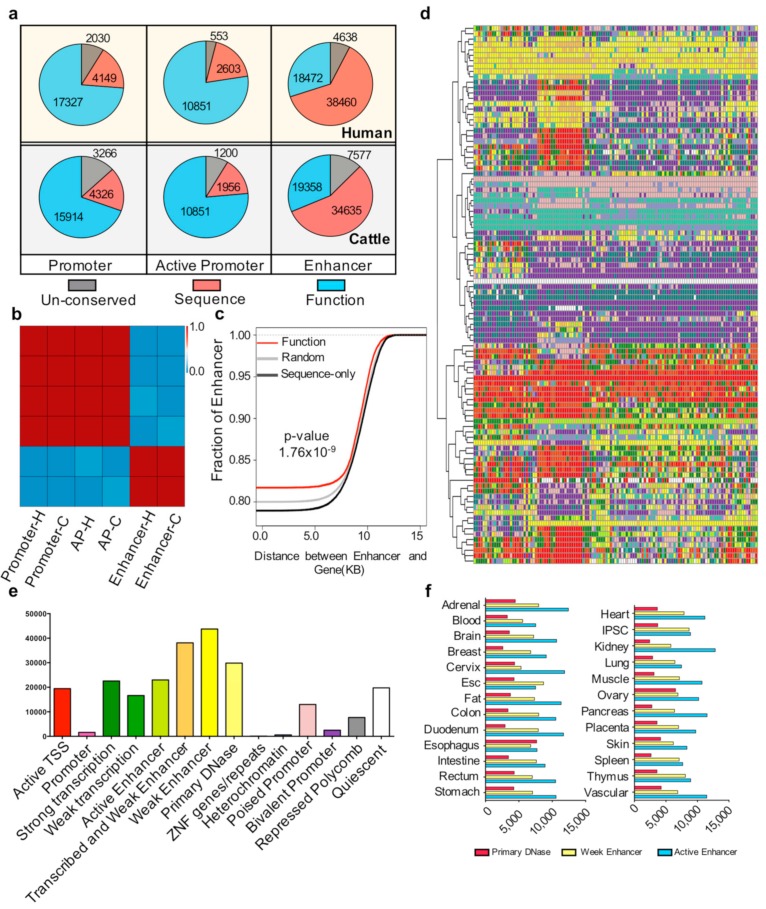
Conservation of *cis*-regulatory elements (**a**) Number of pig un-conserved, sequence-only conserved, and functionally conserved elements with humans and cattle in the promoter, active promoter, and enhancer regions. (**b**) Heatmap of the Pearson correlation coefficient among the three types of conservation state elements. Three types of conservation state elements were un-conserved, sequence-only conserved, and functionally conserved with humans and cattle in the promoter, active promoter, and enhancer regions. H and C indicate pig elements conserved with humans and cattle, respectively. (**c**) Cumulative distribution of the distance of random, sequence only, and functionally conserved enhancers to gene sites. The red, black, and gray lines represent the functionally conserved elements, sequence-only conserved elements, and randomly selected 100 times and selected 4000 elements each time. The *p*-value was determined by the Kolmogorov-Smirnov (KS) test between functionally conserved enhancers and sequence-only conserved enhancers. (**d**) Chromatin states of sequence-only conserved elements in the Roadmap ChromHMM imputation profile. The k-means cluster method (k = 100) was used; each row represents one cluster. For the number of elements in each row and the tissue type in each column, see Supplementary Materials Table S6. The color scale follows the ChromHMM and Roadmap imputation default parameters (Supplementary Materials Table S6). (**e**) Distribution of sequence-only conserved elements that play roles in different chromatin states in the human genome. (**f**) Numbers of primary DNase, weak enhancer, and active enhancer have been imputed in different human tissues.

However, the analysis results of the same comparison of enhancers in pigs, humans, and cattle were very different. More than 87% of pig enhancers were sequence conserved with humans or cattle. However, only 30% and 31.7% of pig enhancers played enhancer roles in humans or cattle, respectively, which were extremely low. These results indicated that a large number of pig enhancers are sequence conserved, but few are functionally conserved in liver tissue of other species (Figure 2a,b).

To explain the characteristics of functionally conserved enhancers further, we investigated the genomic distribution of functionally conserved and sequence-only conserved enhancers. The cumulative results showed that the distance between the functionally conserved enhancers and gene sites is significantly (*p* < 1.76 × 10^−9^) closer than that between sequence-only conserved enhancers and gene sites (Figure 2c). The proportions of sequence-only conserved enhancers are quite different from those of functionally conserved enhancers. Therefore, we determined the role of sequence-only conserved enhancers. We compared the sequence-conserved elements with the Roadmap Epigenetic Projects imputed state [3]. The results showed that most of the sequence-only conserved enhancers (30,073/38,460) can be defined as a primary H3K27ac possible enhancer, active enhancer, or primary DNase in at least one tissue (Figure 2d,e), with the kidney contributing most of these chromatin states (Figure 2f).

### 3.3. Comparison of Promoter States among Pigs, Humans, and Cattle

To investigate the similarities of promoter states among pigs, humans, and cattle, we compared gene expression levels by using RNA-seq [33] (Supplementary Materials Table S7). In total, we identified 1040 significant differentially expressed genes (DEGs) between pigs and humans. Among them, 419 and 621 DEGs showed higher and lower expression levels in the pig liver than in the human liver, respectively (Supplementary Materials Figure S1, Supplementary Materials Table S8). The Gene Ontology (GO) biological process results showed that these DEGs were associated with metabolic related biological processes (Supplementary Materials Figure S2, Supplementary Materials Table S9).

**Figure 3:**
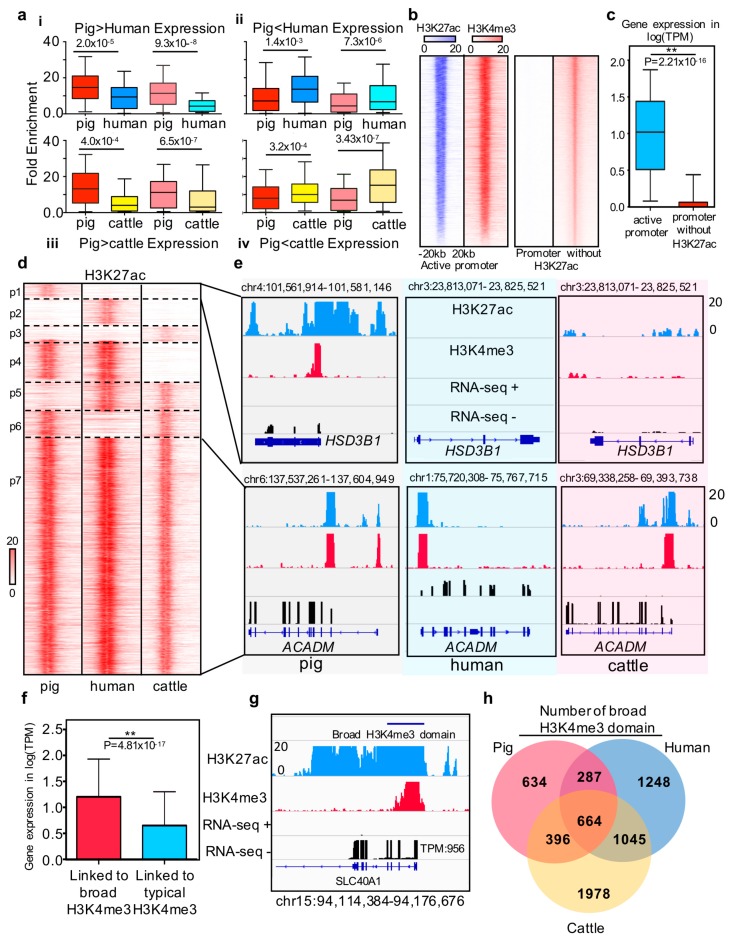
Comparison of promoter states between pigs, humans, and cattle (**a**) Histone intensities for differentially expressed genes between species. (i) and (iii) boxplots show H3K27ac(deep) and H3K4me3(light) intensities for the 419 and 325 genes in which the expression pig is higher than that in humans and cows. The (ii) and (iv) boxplots show H3K27ac(deep) and H3K4me(light) intensities for the 621 and 497 genes in which the expression in pigs is lower than that in humans and cows. Histone intensities and gene expression are quantile normalized in the three species. Statistics analysis was performed using Wilcoxon rank-sum test. (**b)** Heatmap of H3K27ac and H3K4me3 density relative to active promoter and poised promoter region; (**c**) Boxplot of gene expression which linked to the active promoter and poised promoter in pig liver; (**d**) Heatmap of active promoter profile based on H3K27ac signal intensity of each species around the one2one homologous genes. Seven clusters have been marked on the heatmap. The first three clusters represent the genes associated with pigs, humans, and cattle species-specific active promoter, respectively. The fourth to sixth clusters represent the genes associated with cow, pig, and human specific poised promoters, respectively. The last cluster represents the evolutionary stable active promoter in liver tissues across pigs, humans, and cattle. (**e**) Examples of H3K27ac signal intensity of each species around the one2one homologous genes. The up panel represents the RNA-seq, H3K27ac and H3K4me3 profiles of representative pig species-specific active promoters associated with the *HSD3B1* gene and common active promoter associated with the *ACADM* gene. (**f**) Bar plot of expression level of broad H3K4me3 domain and typical H3K4me3 peak linked genes. * represents *p*-value = 4.81 × 10^−17^ (Wilcoxon rank-sum test). (**g**) RNA-seq, ChIP-seq H3K4me3, and H3K27ac profiles of broad H3K4me3 domain located in the *SLC40A1* gene promoter. The expression level of *SLC40A1* is as high as TPM = 956; (**h**) Venn diagram showing the number of functional conserved broad H3K4me3 domain across pigs, humans, and cows.

To explore and extend the chromatin state of these DEGs further, we examined the enrichment intensity of H3K27ac and H3K4me3 histone markers in the 1kb flanking regions of the transcription start sites of DEGs (Figure 3a). The results showed that the 419 DEGs in the pig liver also have higher H3K4me3 and H3K27ac intensity than those in the human liver, and vice versa for the 621 remaining genes. Similar results were obtained in the comparison between pigs and cattle. Four typical examples showed the chromatin state around the DEGs between pigs and humans including *CROT SPP2 MFSD2A* and *THBS1* (Supplementary Materials Figure S3). These results suggested that the intensity of H3K4me3 and H3K27ac corresponds to gene expression levels.

The promoter with H3K27ac is associated with high gene expression levels in humans and mice and is defined as an active promoter [41]. We explored whether this state also exists in pigs, and we determined the feature and regulation function of active promoters among pigs, humans, and cattle. First, we divided 9,499 H3K4me3 binding promoters without H3K27ac signal from the total number of promoters (Figure 3b). Integrated analysis between gene expression and chromatin states indicated that the active promoter-associated genes are generally expressed at higher levels than genes associated with promoters without H3K27ac signal in pigs (*p*-value < 2.21 × 10^−16^ (Figure 3c), as promoter states drive cell development and differentiation [4,14,39]. We next compared the promoter states in three species according to one-to-one homologous genes to explore the similarity of active promoter states among pigs, humans, and cattle (Figure 3d). The results implied three major categories: (i) homologous genes with conserved promoter status, (ii) homologous genes with species-specific active promoter states, and (iii) homologous genes with species-specific promoter with no H3K27ac signal (Figure 3d, Supplementary Materials Table S10). One typical example is *HSD3B1*, which is associated with boar taint and is highly expressed in pig liver tissue [42,43]. In our study, this gene was only expressed in pig liver tissue with species-specific active promoters. Another example is *ACADM*, which is involved in liver lipid metabolism and fatty acid oxidation in mammals [44,45]. In the present study, we confirmed this gene with a stable active promoter in liver tissue across pigs, humans, and cattle.

Moreover, previous studies have reported that the width of H3K4me3 at transcription start sites is associated with a high level of gene expression and can mark cell identity genes and play a powerful role in gene regulation [18,38]. Our results showed a similar phenomenon in pigs: the expression profiling of genes linked to broad H3K4me3 domain is significantly higher than that linked to typical H3K4me3. *SLC40A1* was selected as an example to show the broad H3K4me3 domain with a high expression level in pigs (Figure 3g). To further explore the similarities of broad H3K4me3 domains across pigs, humans, and cattle, we performed conservation analysis to identify the functionally conserved broad H3K4me3 domain. The results showed that 33% of pig broad H3K4me3 domain play a broad H3K4me3 domain role in cattle and human liver tissue. Moreover, 14.5% and 20% of pig broad H3K4me3 domain were shared with humans and cattle, respectively. On the basis of the above results, approximately 68% of the pig broad H3K4me3 domain shared the function in these three species.

### 3.4. Characteristics of Super and Typical Enhancer Crossing Three Mammals

**Figure 4:**
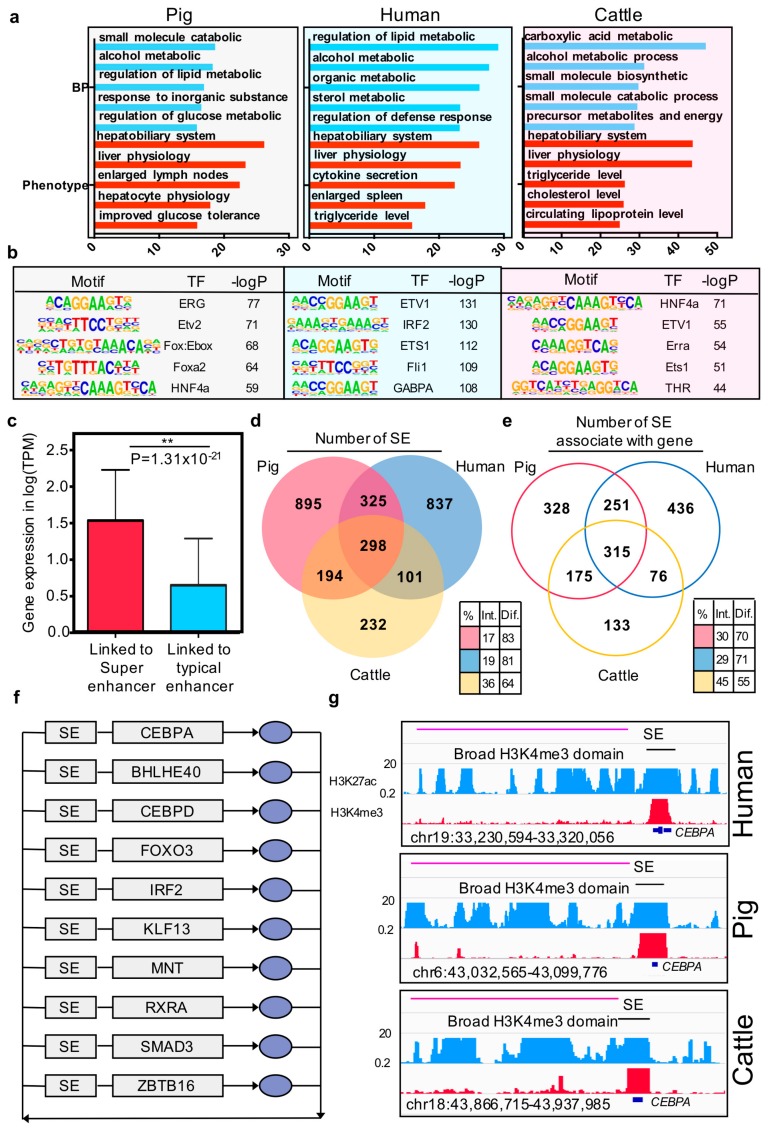
Characteristics of super and typical elements in three mammals. (**a**) Enriched Gene Ontology terms and phenotype terms for the top 3000 enhancers ranked by H3K27ac intensity in pigs, humans, and cows. The *y*-axis represents the –log *p*-value; (**b**) Enriched TF binding motifs with associated *p*-values for top 3000 enhancers in pigs, humans, and cows; (**c**) The bar plot of the gene expression (log TPM) at the linked genes of super-enhancer and typical enhancer. * *p*-value = 4.81 × 10^−17^ (Wilcoxon rank-sum test). (**d**) Venn diagram showing the number of functional conserved super-enhancers across pigs, humans, and cows. (**e**) Venn diagram showing the number of orthologous genes associated with super-enhancers in pigs, humans, and cows. Color-coded tables show the percentages of intersection and difference for each species. The observed differences in intersection between super-enhancer elements and super-enhancer associated with genes in the three species are significant (*p*-values = 1.66 × 10^−16^) based on G-tests of independence. (**f**) Overlapping transcriptional core regulatory circuitry (CRC) cross liver tissues of three species. The grey rectangles represent super-enhancers (SEs) and genes, and the blue oval symbols represent the proteins. (**g**) ChIP-seq, H3K4me3, and H3K27ac profiles of representative conserved broad H3K4me3 domain and super-enhancer located in one of the transcriptional core regulatory circuitry transcription factor *CEBPA* region in the three species.

The similarity of the enhancer is uncertain, and whether they drive the same regulatory function between pigs, humans, and cows has to be further investigated. To address this, we used the web tool Genomic Regions Enrichment of Annotations Tool [46] to identify function terms enriched in the top 3000 ranked enhancer according to H3K27ac intensity from these three species. The biological processes and phenotype enrichment results showed that pig liver enhancers were associated with the metabolic-like processes and hepatobiliary system phenotypes, and the results for humans and cows were consistent with that for pigs (Figure 4a, Supplementary Materials Table S11). Furthermore, the enhancers of pigs were enriched for binding known motifs of hepatic transcription factors (TFs) (Figure 4b, Supplementary Materials Table S12), including ERG, ETV2, FOX, FOXA2, and HNF4A [47,48,49,50,51]. These TFs were also included in human and cow motif enrichment results. These results suggest that enhancers maintain consistency between species in their regulatory patterns.

Previous studies reported that super-enhancers associated with highly expressed genes by enhancing the gene expression. In the current study, we confirmed that, in pigs, the expression value of the gene that was linked to super-enhancers was higher than the gene linked to typical enhancers (*p*-value = 1.31 × 10^−21^, Wilcoxon rank-sum test) (Figure 4c). Further conservation analysis showed that 17% of the super-enhancers in pigs are sequence and functionally conserved in humans and cows (Figure 4d). However, the genes associated with the super-enhancers are the key factor driving cell identity [15]. We then examined the similarity of super-enhancers associated with orthologous genes between the three species. Results indicated that the consistent proportion of genes associated with the super-enhancer was significantly higher than their sequence level across the three species (*p*-value =1.66 × 10^−15^) (Figure 4e).

Core transcription regulatory circuitry (CRC) is an interconnected auto-regulatory loop driven by super-enhancers, the core TF binding to their self, and the other transcription factors in the loop and transcriptional control of cell identify and cellular fate [52]. On this basis, we identified the core transcription regulatory circuitry in pigs, humans, and cattle liver tissues using CRCmapper [35]. Comparison of the CRC results across the three species showed that 10 out of 26 TFs are consistent in all of them, including CEBPA, BHLHE40, CEBPD, FOXO3, IRF2, KLF13, MNT, RXRA, SMAD3, and ZBTB (Figure 4f, Supplementary Materials Table S13). Moreover, these TFs have been reported as key factors in hepatic cells or pioneer factors across developmental processes [53,54,55,56,57]. The liver-specific transcription factor CEBPA was illustrated as an example, which is present in all the core transcriptional regulatory circuitries of the three species, along with a super-enhancer in the gene body region and broad H3K4me3 domain in the transcript start site.

## 4. Discussion

The liver plays a key role in metabolism, especially in lipid and energy metabolism [27]. Liver also affects the feed efficiency of pigs [27,28,29], as well as genes that affect pig growth, such as *IGF2* and *GHR*, are produced mainly in the liver [24,58]. *Cis*-regulatory elements are substantial characteristics of mammalian genome. Early studies showed that enhancer and promoter states are required for transcriptional regulation [4,9,11,13,37]. Increasing number of studies have linked physiological traits and diseases to enhancer and promoter states [10,16]. Given that the pattern of distribution of *cis*-regulatory elements in the genome of livestock is unknown, the annotation of whole-genome *cis*-regulatory elements is very important for understanding and improving the production performance of livestock. In this study, we analyzed the genome-wide H3K4me3 and H3K27ac histone ChIP-seq profile of *cis*-regulatory elements in liver tissue of pigs. Our results identified more than 100,000 *cis*-regulatory in pig liver tissue, including enhancers, active promoters, super-enhancers, and broad H3K4me3 domains. Totally, our *cis*-regulatory annotation in pig liver tissue provides strong background information on pig liver related research. 

During evolution, the conserved non-coding DNA elements are rarely lost during evolution [59]; however, losing conserved elements can affect the phenotypic change, such as the loss of the pelvic spine in spines [60]. Thus, comparing the chromatin state between different species is an effective strategy to improve our understanding of species characteristics and conservation. In this study, we compared the similarity of *cis*-regulatory elements in paralogous species of pigs, humans, and cattle. Early studies have shown many similarities between humans and pigs, such as genome sequence and physiological characteristics of organs [61]. The *cis*-regulatory element annotation of cattle genome, which is another important representative livestock, is also valuable. This comparison is a good model to explore the functional similarity in non-coding regions of pigs, humans, and cattle. As a typical example *HSD3B1* was reported to be associated with boar taint [42,43], in our study, we showed the reason of it being associated with boar taint is probable for its *cis*-regulatory elements. It implies that identification and comparison cross species can improve our understanding of the regulation function of pig genome and also can provide reference data to identify the key genes associated with economic trait of pigs.

Promoters are highly conserved, whereas enhancers are rarely conserved during the evolution [14]. Our results confirmed that promoters more significantly conservative than enhancers in sequence and functional levels. Enhancers exhibit more tissue specificity [4] and play a major role in cell type-specific gene expression during mammalian development [12]. The sequence motifs of TFs are highly similar between species [39]. However, only 1% of liver enhancers are functionally conserved across 20 mammals [14]. The activation of enhancers is dynamic and divergent between pigs and humans, and most of the un-conserved enhancers in liver are activated in other tissues, especially in long-distance regulation. Thus, the sequence conserved enhancer with missing functions in paralogous species liver tissue might be active in other tissues.

Super-enhancers are also conserved across species, and the genes that they regulate are more consistent than their function conserved levels. Most of the core transcription factors in liver tissues are conserved during evolution. The basic functions of liver are highly conserved, and the similarity of the basic functions of hepatocytes in different species is the basic framework of liver tissue function.

Furthermore, the genome-wide association studies provide a large number of markers associated with pig production traits. The mutation such as nonsynonymous that directly affects gene products are receiving attention. However, most of the markers generated from genome-wide association studies identify variants that occur in the non-coding region. Current approaches focus on using the closest gene to the marker or the genes near the maker to speculate on the function of the marker. This often leads to more accurate candidate genes. However, the functional variants which lead to potential regulatory changes cannot be localized. In human studies, RegulomeDB is widely used to locate functional variants. If the variants are located in the *cis*-regulatory element region and caused core-sequence of transcription factor binding motif change, then the variants will be marked by a high score [62]. Our annotation in pig liver also can help provide a hypothetical prediction to the identification of functional variants. For example, recent studies showed that SNP H3GA0002102 (rs80892627) have been identified as significantly associated with Feed conversion ratio (FCR) [63], we note that the +500bp of this SNP has been defined as an enhancer with a strong H3K27ac density in our study (Supplementary Materials Figure S4). This suggests that the probability of functional variants occurring in this enhancer region is very high and this enhancer may be associated with FCR. Therefore, in the future, studying the *cis*-regulatory elements of the pig genome is an efficient way to improve the understanding of results from genome-wide association study related studies of pigs.

Our study described a genome-wide analysis of *cis*-regulatory elements in pig liver and identified the core regulatory transcription factors in this important organ. Moreover, our comparative analyses of *cis*-regulatory elements between pigs, humans, and cattle have enhanced our understanding of the transcriptional regulatory mechanisms of conserved regulatory elements between mammals.

## Supplementary Materials

The following are available online at https://www.mdpi.com/2073-4425/10/5/348/s1, Table S1: The information of ChIP-seq data used in this study, Table S2: Pearson Correlation Coefficient for each ChIP-seq run, Table S3: Number of useful reads and peak for each piece of merged ChIP-seq data, Table S4: List of *cis*-regulatory elements identified in this study, Table S5: List of super enhancers and broad H3K4me3 domain identified in this study, Table S6: List of the tissues/cell line and number of elements within the cluster, Table S7: The information of RNA-seq data used in this study, Table S8: List of DEGs identified in this study, Table S9: Results of functional enrichment in the GO biological process, Table S10: Cluster of different active promoter states between three species, Table S11: GO term for the liver enhancer of three species, Table S12: List of the enriched motifs in enhancer and their enrichment *p*-value, Table S13: List of core transcription factor of liver core regulatory circuitry across three species, Figure S1. The volcano plot of the differential expression genes, Figure S2. The GO term of pig and human differential expression gene. Red bars represent the GO term of the pig higher expression gene, the blue bars present the GO term of human higher expression gene, Figure S3: Example showed differentially chromatin state around DEGs between pig and human, Figure S4: The H3K27ac and H3K4me3 profiles of rs80892627 in ENSSSCG00000004415. The red line represents the rs80892627 position. At the 579bp downstream of rs80892627, a strong and wide enhancer occurred.
